# Exploring the Impact of Guselkumab and Risankizumab on Psoriasis in HIV‐Positive Patients: Insights From Four Italian Centers

**DOI:** 10.1111/ajd.14467

**Published:** 2025-04-03

**Authors:** V. Maione, S. Rovaris, C. Romanó, S. Bighetti, M. Arisi, C. G. Carrera, F. Bellinato, P. Gisondi, Z. Fratton, E. Errichetti, L. Bettolini

**Affiliations:** ^1^ Department of Dermatology, Spedali Civili University of Brescia Brescia Italy; ^2^ Dermatology Unit Fondazione IRCCS Ca' Granda Ospedale Maggiore Policlinico Milan Italy; ^3^ Department of Medicine, Section of Dermatology University of Verona Verona Italy; ^4^ Institute of Dermatology, Department of Medicine University of Udine Udine Italy

**Keywords:** biologics, HIV, psoriasis

## Abstract

HIV‐positive patients with psoriasis often face delays in accessing biologic therapies due to their exclusion from clinical trials and concerns about the impact of immunomodulatory drugs on viral replication. Anti‐IL‐23 therapies, such as risankizumab and guselkumab, have shown great promise thanks to their strong efficacy and favourable safety profiles. A case series from four Italian centres reported sustained effectiveness of these drugs, with no observed effects on viral replication or immune parameters in HIV‐positive patients. Although the number of cases is limited, these therapies appear to be a compelling option for patients with extensive or treatment‐resistant psoriasis.

## 1 Introduction

Psoriasis is a chronic inflammatory skin condition with a prevalence in Human Immunodeficiency Virus (HIV)‐positive individuals comparable to that of the general population. However, these patients often experience more severe disease that is resistant to conventional treatments. Managing psoriasis in HIV‐positive patients requires careful consideration of therapeutic options. First‐line treatments, such as topical agents and phototherapy, are often insufficient due to the increased severity and resistance to treatment observed in this population. Similarly, second‐line options like oral retinoids frequently fail to achieve satisfactory disease control.

Biologic agents have revolutionised the treatment of moderate‐to‐severe psoriasis, offering superior efficacy and safety profiles. However, their use in HIV‐positive patients has been limited and remains underexplored, as these individuals are frequently excluded from clinical trials due to concerns about immunosuppression and the associated risk of infections. Recent evidence, however, suggests that biologics may be a viable therapeutic option for HIV‐positive patients with psoriasis, with anti‐interleukin (IL)‐23 therapies showing particular promise due to their excellent safety profile.

Given the scarcity of available data, this case series aims to evaluate the outcomes of HIV‐positive patients treated with anti‐IL‐23 therapies, focusing on treatment efficacy, changes in HIV replication parameters, and the occurrence of adverse effects.

## 2 Main Text

This retrospective cohort study evaluated the clinical outcomes of HIV‐positive patients undergoing anti‐IL‐23 therapy in real‐world settings at the Dermatology Units of four leading reference centres in Northern Italy for the treatment of psoriasis. Retrospective data collection included the general characteristics of the cohort, the time since HIV diagnosis, and the time since psoriasis diagnosis. Comorbidities and prior treatments were also documented.

For each patient, data were analysed regarding Psoriasis Area and Severity Index (PASI) scores, drug durability, HIV replication rates, and CD4 counts before and after anti‐IL‐23 therapy at Week 16. Adverse drug reactions were additionally recorded. All patients were already receiving Highly Active Antiretroviral Therapy (HAART) at the initiation of anti‐IL‐23 therapy.

The anonymized and encoded database was structured using Microsoft Excel and subsequently imported into IBM SPSS version 27.1 for statistical analysis. Descriptive statistics, including mean, standard deviation, median, and range, were calculated for continuous variables. The Kolmogorov–Smirnov test was used to evaluate the normality of distributions. Non‐normally distributed continuous variables were compared using the paired Wilcoxon test, while normally distributed continuous variables were compared using the independent samples *t*‐test.

Nine patients were enrolled in the study, comprising six men and three women. The main characteristics of the patients are summarised in Table 1. The mean age was 50.89 ± 7.18 years. Among the seven patients, the date of HIV diagnosis was available for only five, with a mean duration since diagnosis of 11.60 ± 7.80 years. Two patients (22%) had concomitant psoriatic arthritis. Ischemic heart disease and HBV co‐infection were reported in 2 (22%) and 3 cases (33%), respectively, while autoimmune conditions such as vitiligo and thyroid disorders were observed in a single patient (11%). Other infections, including HCV or HDV, were not commonly reported.

**TABLE 1:** Principal characteristics of the sample of HIV‐positive patients treated with risankizumab and guselkumab.

Patient ID	Age	Sex	HIV diagnosis age	Psoriasis age	Psoriatic arthritis	PASI before anti IL 23 treatment	PASI 16 week	PASI Week 28	Drug	Drug durability (months)	Previous treatment	CD4 count (cell/ul) before anti IL 23 treatment	CD4 count Week 16	Cd4 count Week 28	HIV replication before anti Il 23 (copy/ml)	HIV replication (copy/mL) Week 16	HIV replication Week 28	Comorbilites	AEs
1	39	M	5	3	No	20	2	1	Risankizumab	11	UVA1	509	472	510	34	19	23	Previous Syphilis	None
2	58	M	11	20	Yes	15	0	0	Guselkumab	24	Apremilast	620	598	709	0	0	0	Chronic Coronary Disease	None
3	59	M	—	31	No	15	1	0	Guselkumab	23	nb‐UVB, Acitretin	N/A	N/A	N/A	64	0	0	Chronic Coronary Disease	None
4	42	M	—	11	No	10.6	0	0	Risankizumab	35	Acitretin	373	388	N/A	0	0	N/A	Vitiligo, Depression, Thyropathy	None
5	59	F	—	20	No	0*	0	0	Risankizumab	10	Acitretin, Adalimumab	647	921	N/A	0	30	N/A	HBV	None
6	52	F	—	29	No	13	0	0	Risankizumab	6	Methotrexate	1300	1390	N/A	0	0	N/A	Hypercholesterolemia	None
7	48	M	23	37	Yes	16	3	0	Risankizumab	12	Etanercept, Ustekinumab	370	280	310	0	0	0	HCV, HBV, HDV	None
8	50	M	15	31	No	15	0	0	Risankizumab	6	nb‐UVB	990	980	960	0	0	0	HBV	None
9	51	F	4	12	No	12	1	1	Guselkumab	8	Acitretin	412	448	430	19	10	10	None	None

In 67% of cases, the chosen therapy was risankizumab, while guselkumab was prescribed in the remaining 33%. Prior to initiating anti‐IL‐23 therapy, the most commonly prescribed treatments included acitretin (44%) and ultraviolet light therapy (33%, UVA1 or nb‐UVB). Only two patients had previously been treated with TNF‐α (22%) inhibitors or IL‐12/23 inhibitors (11%).

**FIGURE 1:**
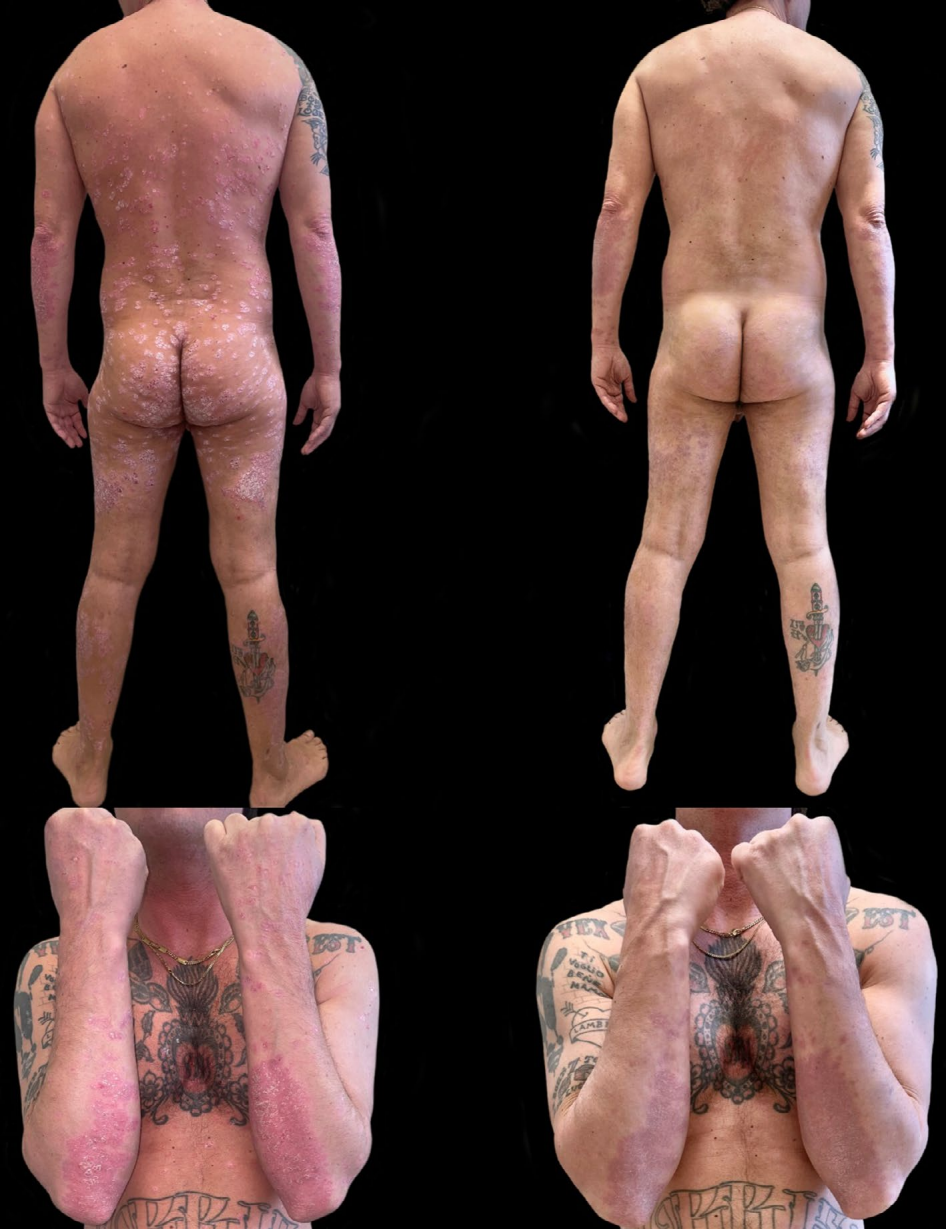
Patient treated with Risankizumab achieved a PASI 100 at Week 28 of therapy. During treatment, fluctuations in CD4 replication rates and viral load were observed without affecting the efficacy of antiretroviral therapy.

The median PASI score at treatment initiation was 15 (interquartile range (IQR):0–20). One patient had a baseline PASI score of 0 at the start of anti‐IL‐23 therapy; the switch to this therapy was motivated by the need for longer dosing intervals, as the patient lived far from the prescribing center. By Week 16, a significant reduction in PASI was observed, with a median score of 0 (IQR: 0–3), which further improved by Week 28 to a median of 0 (IQR: 0–1). These results demonstrated a statistically significant improvement with both drugs from baseline to Week 16 (*z* = −2.52, *p* = 0.008), with a sustained response observed from baseline to Week 28 (*z* = −2.52, *p* = 0.008). PASI 100 was achieved in 55.56% of cases, and PASI 90 in 88.89% at Week 16. By Week 28, PASI 100 was achieved in 77.78% of cases, with PASI 90 reached in 100% of cases (Figure 1).

The drug survival rate for both therapies had a median duration of 11 months (IQR: 6–35 months) by the end of the data collection period. No statistically significant differences were observed in CD4 counts before and after the introduction of anti‐IL‐23 therapy at each follow‐up visit (*t* = −0.56, *p* = 0.6 and *t* = −1.18, *p* = 0.30, respectively). Notably, CD4 values remained within the normal range throughout the study. Similarly, no significant impact was observed on viral replication rates, which, despite minimal fluctuations, consistently remained undetectable throughout each follow‐up visit (*z* = 0.0, *p* = 1.00 and *z* = −1.34, *p* = 0.32, respectively).

No drug‐related adverse events were reported during the treatment period.

## 3 Conclusion

The treatment of psoriasis in HIV‐positive patients presents a therapeutic challenge. The introduction of HAART has significantly impacted the virus's replicative capacity, leading to notable benefits such as increased CD4 counts and reduced progression to full‐blown Acquired Immunodeficiency Syndrome (AIDS) [1]. However, these benefits could potentially be compromised by the use of immunosuppressive drugs, which are commonly first‐line treatments for psoriasis [2]. For example, methotrexate, a well‐known drug for the treatment of psoriasis, does not seem to alter HIV replication but leads to a reduction in CD4 levels [3]. For this reason, the use of selective immunomodulators, such as biologic therapies, appears more advantageous. Nevertheless, the literature on the use of these drugs in this special population is limited and primarily consists of case series.

Current evidence predominantly highlights the use of anti‐TNF alpha agents, such as etanercept or adalimumab, and anti‐IL‐12/23 agents, such as ustekinumab. Although the sample sizes are small, these agents do not seem to interfere with the virus's replicative capacity during HAART. A recent systematic review of the literature analysing the rate of viral reactivation during biologic therapy suggests a possible, albeit low, rate of HIV reactivation [4]. However, among the studies included in the review, no cases of reactivation were observed. It is important to note that only two studies were analysed, comprising a total of 13 patients [5, 6]. On the other hand, a few case reports have demonstrated that anti‐IL‐17 agents are a safe option for treating psoriasis and psoriatic arthritis in HIV patients [7, 8].

The recent introduction of IL‐23 pathway inhibitors offers an additional therapeutic option. Their high selectivity and efficacy have led to their rapid adoption in the dermatologic therapeutic arsenal. Moreover, their reduced propensity to induce infections makes them particularly suitable for certain at‐risk populations. For example, preliminary data suggest that some of these agents may not reactivate latent tuberculosis [9]. Regarding HIV‐positive patients, the literature includes a few recent studies. For instance, Orsini et al. report four cases of HIV‐positive patients treated with risankizumab, demonstrating significant improvement in both the PASI and the Dermatology Life Quality Index (DLQI) [10]. However, this study does not provide data on viral dynamics or changes in CD4^+^ counts. Other individual case reports document the use of risankizumab and guselkumab in HIV‐positive patients [11, 12].

A more comparable study is the recent work by Xu et al., which analysed 36 HIV‐positive patients treated with various biologic agents, including seven treated with risankizumab [13]. Among these patients, 100% achieved PASI 75, 87.5% achieved PASI 90, and 57.7% achieved PASI 100 at 12 month of therapy. In our study, all patients treated with an IL‐23 inhibitor achieved PASI 90 at 28 weeks, and a higher percentage achieved PASI 100. Additionally, the authors of Xu et al.'s study evaluated CD4^+^ counts and HIV replicative rates during treatment in psoriasis patients compared to HIV‐positive patients without psoriasis. Although some fluctuations in CD4^+^ counts and replication rates were observed, the values in psoriasis patients did not differ significantly from those in the control group. Similar findings were confirmed for viral replicative rates. These results align with our collection, although our study lacked a comparison group.

Notably, no infectious complications were reported among patients treated with anti‐IL‐23 agents in the analysed case series.

In conclusion, the treatment of psoriasis in HIV‐positive patients requires a delicate balance between managing the inflammatory condition and maintaining effective immune control of the virus. While biologic therapies, particularly IL‐23 inhibitors, show promising efficacy and safety profiles in this population, the limited available data underscores the need for further research. Larger, controlled studies are necessary to confirm these findings, better characterise long‐term outcomes, and establish standardised guidelines. In the interim, a multidisciplinary approach, involving dermatologists, infectious disease specialists, and immunologists, remains crucial to optimise patient care and ensure that both psoriasis and HIV are effectively managed without compromising patient safety.

## Author Contributions

All authors contributed equally to this work. Conception V.M. Design V.M., S.R., C.R., S.B., and L.B. Acquisition of data V.M., S.R., M.A., C.G.C., F.B., P.G., Z.F., and E.E. Analysis and interpretation of data V.M., S.B., and L.B.

## Consent

The patients in this manuscript have given written informed consent to publication of their case details.

## Conflicts of Interest

The authors declare no conflicts of interest.

## Data Availability

The data that support the findings of this study are available from the corresponding author upon reasonable request.
